# Dense Pedestrian Detection Based on GR-YOLO

**DOI:** 10.3390/s24144747

**Published:** 2024-07-22

**Authors:** Nianfeng Li, Xinlu Bai, Xiangfeng Shen, Peizeng Xin, Jia Tian, Tengfei Chai, Zhenyan Wang

**Affiliations:** College of Computer Science and Technology, Changchun University, No. 6543, Satellite Road, Changchun 130022, China; 231501498@mails.ccu.edu.cn (X.B.); 210701252@mails.ccu.edu.cn (X.S.); 230702299@mails.ccu.edu.cn (P.X.); 230702297@mails.ccu.edu.cn (J.T.); ch17861525698@163.com (T.C.); 220701275@mails.ccu.edu.cn (Z.W.)

**Keywords:** target detection, pedestrian detection, Yolov8

## Abstract

In large public places such as railway stations and airports, dense pedestrian detection is important for safety and security. Deep learning methods provide relatively effective solutions but still face problems such as feature extraction difficulties, image multi-scale variations, and high leakage detection rates, which bring great challenges to the research in this field. In this paper, we propose an improved dense pedestrian detection algorithm GR-yolo based on Yolov8. GR-yolo introduces the repc3 module to optimize the backbone network, which enhances the ability of feature extraction, adopts the aggregation–distribution mechanism to reconstruct the yolov8 neck structure, fuses multi-level information, achieves a more efficient exchange of information, and enhances the detection ability of the model. Meanwhile, the Giou loss calculation is used to help GR-yolo converge better, improve the detection accuracy of the target position, and reduce missed detection. Experiments show that GR-yolo has improved detection performance over yolov8, with a 3.1% improvement in detection means accuracy on the wider people dataset, 7.2% on the crowd human dataset, and 11.7% on the people detection images dataset. Therefore, the proposed GR-yolo algorithm is suitable for dense, multi-scale, and scene-variable pedestrian detection, and the improvement also provides a new idea to solve dense pedestrian detection in real scenes.

## 1. Introduction

Pedestrian detection has always been a prominent research direction in target detection. In densely populated public places such as railway stations and airports, accurate pedestrian detection is crucial for ensuring public safety. It can promptly detect potential safety hazards and provide an important basis for the reasonable allocation of traffic flow and corresponding security measures. The core task of pedestrian detection is to identify all the pedestrians in an image or video frame, regardless of their location and size, with the target annotation generally being a rectangular box. In addition to pedestrian detection, target detection also covers typical problems such as face detection, vehicle detection, and remote sensing detection.

Pedestrian detection technology has significant application value. It can be combined with pedestrian tracking [1], pedestrian re-identification [2], and other technologies, and applied to unmanned systems [3], intelligent transportation [4], intelligent robotics [5], intelligent video surveillance [6], human behavior analysis [7], and other fields. In particular, dense pedestrian detection is crucial for large public places such as railway stations and airports, where the flow of people in dense areas directly affects the distribution of traffic flow and the corresponding security measures.

Pedestrian detection faces several challenges due to the diversity of human postures and the vast differences in appearance at different angles, lighting conditions, and levels of occlusion. For example, occlusion between pedestrians can lead to difficulties in feature extraction, making it challenging for the model to accurately identify the location and number of pedestrians.

Based on the principles of algorithm implementation, pedestrian detection algorithms can be classified into two types: stationary detection-based algorithms and deep learning-based algorithms. Stationary detection-based algorithms assume that the camera is stationary. They utilize background modeling algorithms to extract foreground targets in motion. A classifier is then used to classify these moving targets and determine if they contain pedestrians. Classical foreground modeling algorithms include the Gaussian mixture algorithm, VIBE algorithm, frame difference algorithm, and sample consistency algorithm.

The development of target detection based on deep learning can be divided into two cycles. The first cycle is based on the traditional manual extraction of target detection algorithms. Despite the long period of development, traditional target detection algorithms have not significantly improved in recognition effectiveness. They also require substantial computational resources and have gradually faded from the forefront of target detection research.

The second cycle is based on deep learning target detection algorithms, which are primarily categorized into two-stage and one-stage approaches. In the two-stage approach, candidate box regions likely to contain the target are first generated. Then, feature extraction is performed on these candidate regions, followed by sample classification using convolutional neural networks (CNNs). Common two-stage methods include R-CNN [8], which pioneered the application of deep learning to image recognition tasks, and its derivatives such as faster R-CNN [9] and fast R-CNN [10]. These algorithms offer advantages in detection accuracy but suffer from long training times and slow inference speeds. On the other hand, one-stage approaches directly treat target detection as a regression task across the entire image without generating candidate boxes. Representative one-stage algorithms include the Yolo series and the single-shot multi-box detector (SSD) [11]. One-stage algorithms reduce training time and accelerate inference speed but may sacrifice some accuracy.

Although target detection techniques have made significant progress through traditional and deep learning methods, there is still relatively less research on dense pedestrian detection in the presence of substantial occlusion. The accuracy and speed of dense pedestrian detection still require further improvement, leaving ample room for enhancement in multi-scene and dense-scene pedestrian detection. Deep learning methods have achieved certain results in pedestrian detection, yet they face significant challenges in dense pedestrian scenarios. The high data complexity in dense pedestrian scenes and the mutual occlusion between pedestrians make feature extraction exceptionally challenging. Datasets like the crowd human dataset from the Crowd Vision Research Institute and the broader person dataset curated by the Biometrics and Security Research Centre at the National Pattern Recognition Laboratory, Institute of Automation of the Chinese Academy of Sciences, provide multi-scene images with varying degrees of pedestrian density. These datasets play a crucial role in advancing research on dense pedestrian detection. The broader person dataset, in particular, extends beyond just traffic scenes to encompass a wide range of scenarios, marking a significant step towards enhancing pedestrian detection technology for dense environments. This advancement holds substantial practical significance for fields such as autonomous driving and security monitoring. Moreover, issues such as multi-scale variations in images and high false positive rates also severely impact the accuracy and reliability of detection.

Aiming at addressing issues such as low detection efficiency, difficulty in feature extraction, image multi-scale variation, and high false detection rate due to pedestrian occlusion or dense crowd flow in crowded areas, this paper proposes a new detection model, GR-YOLO (Gold-Repc3 YOLO), based on YOLOv8n to improve the aforementioned challenges. The specific contributions of this paper are outlined in the following four aspects:

(1) To tackle the dense pedestrian detection challenges arising from pedestrian occlusion, where backgrounds are often misclassified as pedestrians, the Repc3 module is introduced to optimize the backbone network of YOLOv8. The Repc3 module enhances feature integration and information enhancement through convolution operations, thereby improving the model’s feature expression capability and addressing issues with feature extraction due to pedestrian occlusion.

(2) The paper introduces an aggregation–distribution mechanism that represents a paradigm shift aimed at enhancing model perceptual discrimination through multi-scale feature fusion. This mechanism efficiently exchanges information in YOLOv8 by fusing features across multiple layers and injecting global information into higher layers. This enhancement significantly boosts the fusion capability of the network’s neck architecture, thereby improving the model’s final detection performance and addressing challenges related to insufficient feature fusion capabilities for multi-scale image variations.

(3) In scenarios involving frequent occlusion of target objects, model convergence may be slower, and there could be instances of missed detection. To mitigate these issues, the paper employs the Giou loss function, which aids model convergence and is particularly sensitive to detecting overlapping situations. This approach enhances the model’s accuracy in predicting target locations and reduces missed detections when dealing with dense data.

(4) The effectiveness of the proposed algorithm is validated through comprehensive multi-group ablation experiments and comparisons with mainstream target detection algorithms. These experiments analyze final results and provide significant advancements in the field of dense pedestrian detection.

These contributions collectively propel advancements in dense pedestrian detection, addressing critical challenges in crowded environments and scenarios with mutual occlusion.

## 2. Related Work

### 2.1. Literature Review

In recent years, pedestrian detection has become a popular research aspect in the field of target detection, and a large number of research results have emerged. Zhida Huang [12] and others proposed a visible feature bounding box mechanism, which improves the performance of the detector using visible feature regression, visible bounding box, and complete bounding box simultaneous outputs during the training and inference process, but the detection is not good in a dense pedestrian dataset. Based on the fact that detection in crowded and complex scenes is plagued by target detection and localization, Thittaporn Ganokratanaa [13] et al. proposed a novel unsupervised anomaly localization method based on generative adversarial networks and edge wrapping, deep spatio-temporal torsion networks, which was tested on publicly available anomaly datasets and shown to outperform other algorithms, but also showed limited improvement in detection accuracy for datasets with multiple scenes and irregular scale sizes. Wen-Hui [14] et al. proposed a lightweight, high-performance edge computing solution to reduce the leakage rate of yolo-tiny from 48.8% to 26.2%, but the accuracy rate also decreased. Songtao Liu [15] et al. designed an efficient sub-network with a novel, non-extremely large suppression algorithm to better refine the bounding box provided by the detector learning density scores to achieve advanced results on the benchmarks of the city persons dataset and crowd human dataset. Yanwei Pang [16] and others proposed a novel mask-guided attention network for the occluded pedestrian detection method, which suppresses the occluded region by adjusting the whole-body features, effectively copes with the intra- and inter-class occlusion problem in pedestrian detection, and improves the accuracy of dense pedestrian detection. XiaoLin Song [17] and others designed a novel single-stage detector that introduces a full-body template based on occlusion statistics and a confidence-aware calibration process, which effectively mitigates the problem of occluded pedestrian detection through three-stage gradual refinement. Yongjun Li [18] et al. proposed an efficient detector YOLO-CAN, which improves the detection accuracy of small targets and occluded objects by introducing improvements such as the attention mechanism, the Ciou loss function, soft-nms, and depth-separable convolution, but there is no improvement in the detection efficiency of the occlusion problem present in dense pedestrian datasets. Jialiang Zhang [19] and others proposed an attribute-aware pedestrian detector that can effectively distinguish individuals in crowded scenarios and alleviate the difficulties of traditional detectors in detecting dense environments through the introduction of attribute features and attribute-based non-maximal suppression algorithms. Wei-Yen Hsu [20] and others proposed a method called ratio-aware yolo that reduces the problems caused by differences in pedestrian ratios and input image angles. Hexiang Zhang [21] et al. proposed a high-density pedestrian detection algorithm based on deep information fusion, which further integrates high-level semantic information and feature information by increasing the connecting points of cross-layer fusion, the improved anchor values are more adapted to the network model, and the network anti-jamming ability is enhanced by replacing the Ciou target detection object. Xiaxia Zhang [22] et al. proposed an improved lightweight network MobileNetv3 model based on Yolov3. Firstly, the improved MobileNetv3 replaces Darknet53 for feature extraction to reduce the complexity of the algorithm and for model simplification. Secondly, the complete IOU containing an overlapping region, the center of mass distance, and aspect ratio is introduced. Secondly, a complete IOU, including overlapping region, center-of-mass distance, and aspect ratio, is introduced to enable the model to achieve faster convergence and better performance. In addition, a new attention module, SESAM, is constructed to improve the model detection performance at a long distance using the channel attention and spatial attention in MobileNetv3, but there is no enhancement of the detection accuracy in complex scenes and dense regions. Currently, most of the research methods generally have low accuracy for pedestrian detection in dense scenes, and it is difficult to balance the relationship between model accuracy and leakage rate.

### 2.2. Model Introduction

Yolo is a real-time target detection system that has undergone several optimizations and updates since its first release. Yolov1 first treated the target detection problem as a single regression problem by predicting bounding box and category probabilities directly from the image via a neural network. Yolov2 introduced improvements to Yolov1, such as multi-scale pre-batch normalization, high resolution, and anchor frames, improving accuracy and speed. Yolov3 further improves the network architecture by introducing residual networks, multi-scale feature pyramids, and better loss functions, which improve the detection accuracy while maintaining the speed advantage. Yolov4 introduces many modern techniques for target detection, such as the cspdarknet53 backbone network, the mish activation function, the feature pyramid, and the path-aggregation network, which significantly improve the accuracy and speed of the model. Yolov5 adopted the Pytorch framework and utilized the CSP-net structure as the backbone network, which improved the accuracy and speed of detection. Yolov6 further optimized the network structure and training strategy, focusing on improving the accuracy and speed of detection. Yolov7 introduced a new training strategy and network structure, which further improved the accuracy and speed. Yolov8 introduced new features and improvements to further enhance performance and flexibility, including a new backbone network, a new anchor-free detection header, and a new loss function that focuses on maintaining the optimal balance between accuracy and speed. yolov9 introduced the concept of programmable gradient information to cope with the variety of variations required for deep networks to achieve multiple goals. yolov10 is the next generation of the YOLO family for real-time end-to-end target detection, which achieves NMS-free training by introducing a consistent dual allocation strategy.

### 2.3. Standard YOLOv8

Yolov8 is a newer iteration of the Yolo series of real-time object detectors, introducing new features and improvements to the previous Yolo series to enhance detection performance and flexibility, making it the best choice for a wide range of target detection tasks. However, the official name is not “yolov8” but “ultralytics” because “ultralytics” is positioned as an algorithmic framework, not just a specific algorithm. An important feature is that it can be used not only for the Yolo family of models but also for non-yolo family models and other image tasks such as segmentation, detection, pose evaluation, etc.

**Figure 1:**
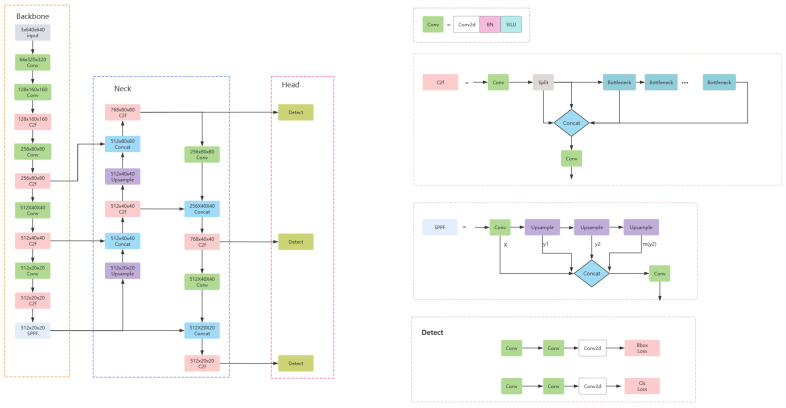
Yolov8 model structure diagram.

Yolov8 provides a newer state-of-the-art (SOTA) model and offers different scale sizes for different scenario requirements. It is also divided into three main parts: the backbone, neck, and head networks. The backbone network for feature extraction and the neck network for feature fusion are designed concerning the yolov7ELAN idea, and the C3 structure in the yolov5 backbone is replaced by the C2f module, which has fewer parameters, stronger adaptive ability, and faster computation speed, so that modules with no need for scale size can adapt to a different number of channels, which can improve the detection performance of the model significantly. The head network part uses the current mainstream decoupled head structure to separate the classification and detection heads, which improves the flexibility of the model, reduces the computational cost, and makes it easier to expand. At the same time, the anchor-based method is replaced by an anchor-free method, which aims to reduce the associated computation, design complexity, and hyper-parameters, as well as to improve the detection accuracy. Yolov8 focuses on maintaining the optimal balance between accuracy and speed and is suitable for real-time target detection tasks in a variety of application areas. The model structure diagram is shown in Figure 1.

## 3. Overview of the Dense Pedestrian Detection Model

### 3.1. Introduction to the Datasets

#### 3.1.1. WiderPerson Dataset

WiderPerson [23] is a common benchmark dataset for outdoor pedestrian detection. The dataset scenarios are not limited to traffic scenarios but also cover other life scenarios where there are large numbers of dense crowds, such as playgrounds, studios, stations, etc. The WiderPerson dataset contains a total of 13,382 images labeled with 400,000 different occlusion targets, of which 8000 images are used for training, 1000 images for validation, and 4382 images for testing.

**Figure 2:**
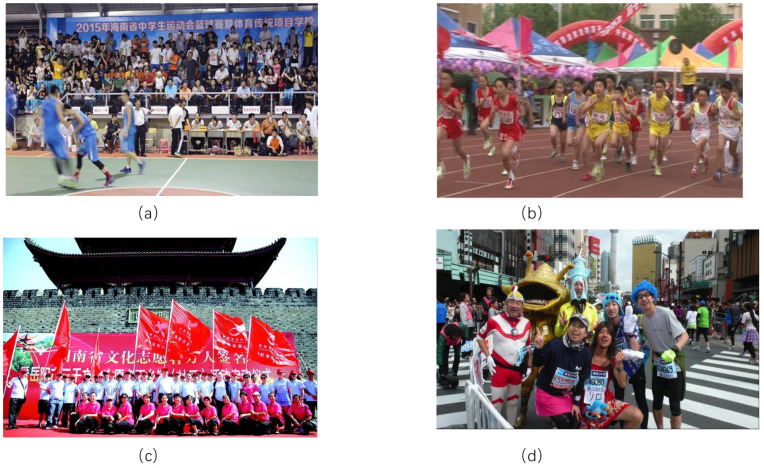
WiderPerson data set: (**a**–**d**) Large Crowds of People Living Scene.

Due to equipment limitations, 7199 images are randomly selected as the training set, 901 images as the validation set, and 775 images as the test set in this experiment. The images shown below are four randomly selected images from the WiderPeople dataset, which show dense pedestrians in different scenarios. A sample dataset is shown in Figure 2.

#### 3.1.2. CrowdHuman Dataset

**Figure 3:**
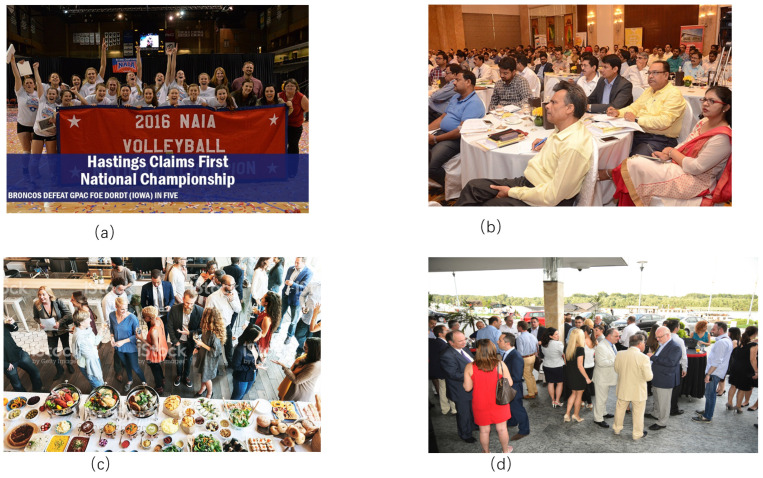
CrowdHuman data set: (**a**–**d**) Dense pedestrian detection baseline.

The CrowdHuman dataset [24] is a dedicated benchmark dataset for dense pedestrian detection released by the Kuangxiang Research Institute. The dataset is large, richly annotated, and contains complex and diverse image representations. The CrowdHuman dataset contains 15,000 images for training, 4370 images for validation, and 5000 images for testing. The entire dataset contains 470,000 instances of pedestrians, and a single image in the dataset contains an average of 23 people, with a variety of occlusions in the photos. The figure below shows four randomly selected images from the CrowdHuman dataset, where (a), (b), and (d) are more occlusion scenarios and (c) is less occlusion. A sample dataset is shown in Figure 3.

#### 3.1.3. People Detection Image Dataset

**Figure 4:**
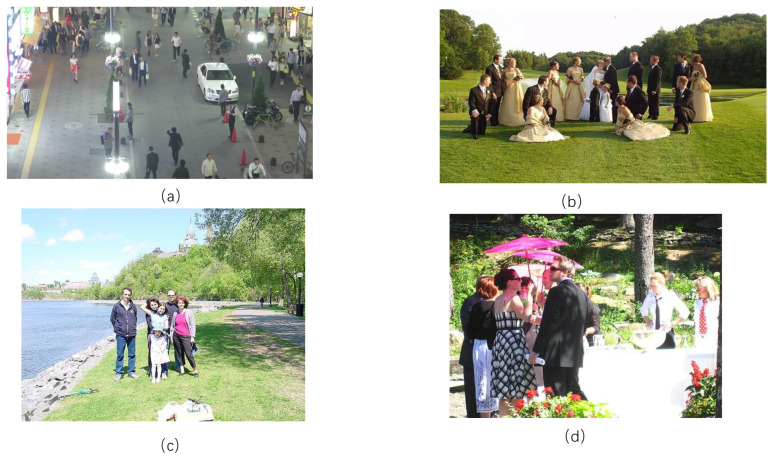
People Detection Image data set: (**a**–**d**) Crowd detection in various scenarious.

The People Detection Image Dataset, from the roboflow platform officially released by Ultralytics, is a crowd detection dataset for generalized detection of various scenarios [25], which covers the diversity of crowd densities in different scenarios. Specifically, the dataset contains a total of 15,210 images for training, 1431 images for validation, and 760 images for testing. The four photos shown in the figure below are four randomly selected photos from the people detection dataset, and the four photos show different scenarios, different crowd densities, and different levels of occlusion. A sample dataset is shown in Figure 4.

#### 3.1.4. Data Preprocessing

Dense datasets are larger in size and rich in labeled information compared to other datasets and are labeled at multiple scales, covering a wide range of different crowd densities, levels of occlusion, and complex background information. The highly diverse data distribution makes it more challenging to perform detection compared to other datasets. Moreover, when detecting crowded data, the occlusion problem in the crowd must also be taken into account, and the dataset mentioned above provides an effective means to deal with the occlusion problem through fine labeling and attribute information.

The image preprocessing scheme of feature-based data filtering makes it possible to leave only the bounding box information of the pedestrian full-body annotation for the head bounding box, the human visible area annotation box, and the pedestrian full-body bounding box annotated in the crowd human dataset. For the pedestrian labeled box, the cyclist labeled box, the partially visible human labeled box (pedestrians are heavily occluded), the dense crowd labeled part, and the ignored region included in the wider people dataset, the data are preprocessed so that only one category of pedestrians is left. This allows for better training and prediction of subsequent models for dense pedestrian detection.

### 3.2. Improved GR-YOLO

In this paper, a dense pedestrian detection model with both improved detection accuracy and reduced leakage rate is constructed from the following three aspects:

Firstly, to address the problem of low feature extraction capability due to pedestrian occlusion in dense pedestrian detection, the Repc3 module is introduced to optimize the backbone network and improve the feature extraction capability. The Repc3 module is able to better capture the feature information of occluded pedestrians through more efficient feature representation.

Secondly, for the problem of image multi-scale changes, the fusion aggregation–distribution mechanism is used to reconstruct the neck structure, which fully takes into account more comprehensive and advanced multi-scale feature fusion. Through this mechanism, the model is able to better handle pedestrian targets at different scales and improve the robustness and accuracy of detection.

Finally, Giou loss is used to address the problem of the high miss-detection rate in pedestrian detection. Giou loss is more sensitive to detecting overlapping situations, which improves the accuracy of the model in predicting the target location and reduces the occurrence of miss detection. With these improvements, the model shows a higher detection accuracy and lower pickup rate in dense pedestrian detection tasks.

#### 3.2.1. Backbone Optimization

In dense pedestrian detection, occlusion between pedestrians often leads to feature extraction difficulties, which negatively affect the detection results. To effectively deal with this problem, this paper introduces the RepC3 module to optimize the backbone network, which has unique advantages in feature extraction, making it better adapted to the complex scenarios of dense pedestrian detection. Specifically, the RepC3 module performs feature extraction through multiple RepConv layers, each of which contains a 3 × 3 convolutional kernel and a 1 × 1 convolutional kernel. The 3 × 3 convolutional kernel can capture local features for detailed feature extraction from the input image, while the 1 × 1 convolutional kernel is responsible for integrating and downscaling features, which reduces the amount of computation while retaining important information. This combination allows the model to extract more complex and rich features while keeping the complexity low, thus better coping with the complexity and variability of pedestrian poses and occlusion in dense pedestrian scenes.

In addition, the attention mechanism inside the RepC3 module can dynamically assign weights according to the importance of the data in the input image. During the detection process, this mechanism enables the model to pay more attention to the key parts where pedestrian targets exist, especially in the case of severe pedestrian occlusion, and can precisely focus on the key features that are not occluded, significantly improving the accuracy of target detection. Meanwhile, the residual connection used in the RepC3 module helps to deepen the number of network layers and enhance the network’s ability to learn data features. In dense pedestrian detection, this deep network learning ability enables the model to understand the occlusion relationship between pedestrians more deeply and then extract more targeted and effective features, which greatly improves the model’s feature extraction ability and detection accuracy in complex environments.

**Figure 5:**
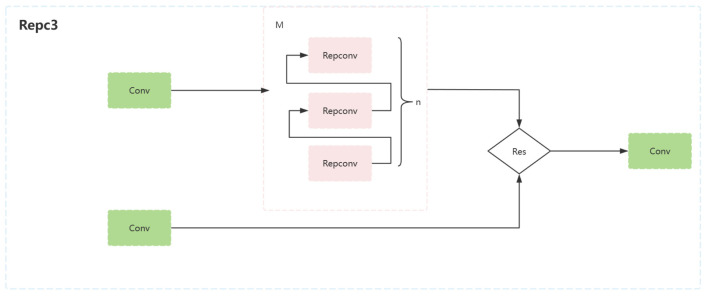
Repc3 model structure diagram.

In dense pedestrian scenarios, the attention mechanism and residual connectivity of the RepC3 module enable the model to better cope with the challenges posed by pedestrian occlusion, accurately extract features of pedestrians, and reduce the leakage detection rate. Thus, the backbone network is optimized to provide richer features for the neck for further scale fusion, which makes it perform better in the dense pedestrian detection task and improves the detection performance of the whole model. In summary, the RepC3 module, through its unique structure and function with good adaptability, can effectively solve the difficult feature extraction problem in dense pedestrian detection and makes an important contribution to improving the detection performance of the model and solving the dense pedestrian detection problem. The model structure diagram is shown in Figure 5.

#### 3.2.2. Neck Reconstruction

The Yolo family of models has become the preferred solution for target detection due to its excellent performance. Currently, many studies have improved the baseline of this model to a high level by modifying the framework of the backbone network [26], optimizing the modules in the backbone network [27], designing and improving the new attention module [28], and optimizing the detection head [29].

The FPN + PAN structure adopted by the original yolov8 is an optimized update of the FPN structure, but it has some problems in the actual feature scale fusion process. Specifically, the main role of FPN is to transfer the underlying information features to the higher layers to enhance the semantic representation on multiple scales. However, in dense pedestrian detection applications, the lack of sufficient attention to large-scale feature maps in this structure may cause the model to ignore the useful information embedded in some underlying features, which reduces the detection quality. In addition, the underlying information needs to be passed through multiple layers to reach the higher layers, during which the underlying features will lose some information after long upsampling and downsampling paths, which will also increase the computation and model complexity. Based on this, it is necessary to reconstruct the neck structure for dense pedestrian detection models.

**Figure 6:**
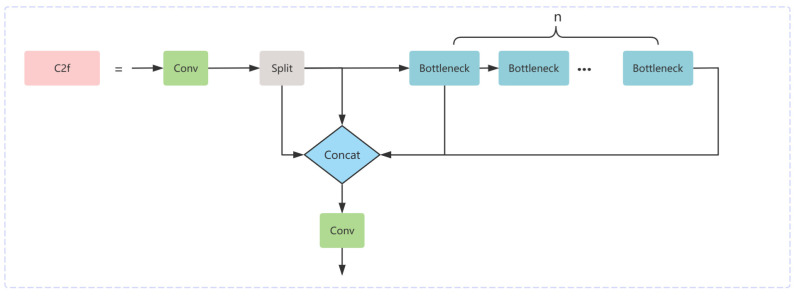
C2f model structure diagram.

Firstly, in order to increase the focus on large-scale feature maps, an upsampling process is added to the FPN and makes it deeply fused with the underlying information in the backbone network as a way to improve detection accuracy. Unlike the upsampling process of previous scale fusion structures, this improvement uses the C2f module and the Repc3 module for further feature extraction fusion. The C2f module consists of two 1 × 1 convolution kernels and a series of bottleneck modules, where the convolution serves to perform feature extraction and transformation on the input image data. In contrast, the bottleneck is used by C2f as the output of the intermediate layer and can either be fused directly with the feature maps of other layers or fused with the feature maps of other layers after upsampling. This improvement effectively enhances the model’s ability to detect targets at different scales, and its specific structure is shown in the following Figure 6.

Secondly, to optimize the feature transfer path, the idea of an aggregation–distribution mechanism [30] is introduced. This mechanism improves the ability of multi-scale fusion through a self-attention module and convolution operation, which achieves the best balance of latency and accuracy at all model scales and significantly reduces the problem of information loss in the process of transferring information from the bottom layer to the top layer. The aggregation–distribution mechanism consists of three main parts: the feature alignment module (FAM) is responsible for collecting feature map information aligned from different layers; the information fusion module (IFM) is used to perform feature fusion of this aligned information to obtain global feature information; the information injector module (Inject) injects global information collected from the FAM into different layers of the model, thus achieving efficient information extraction and fusion. With these improvements, the new neck structure is better able to handle dense pedestrian detection tasks and improve the overall performance and efficiency of the model.

**Figure 7:**
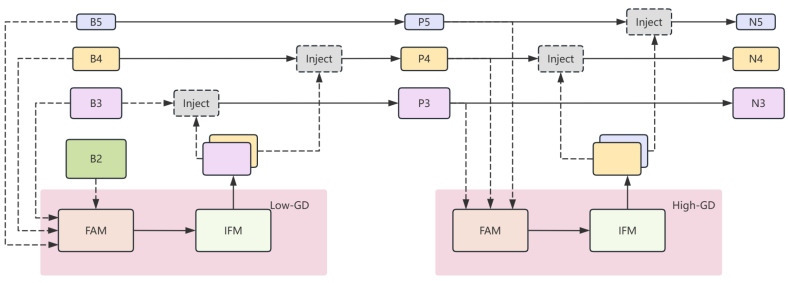
Aggregation–distribution mechanism in the yolov8 application structure diagram.

In yolov8, according to the feature map size in ascending order, is divided into five kinds of scale features, which are denoted as B5, B4, B3, B2, and B1 in the backbone network, P5, P4, and P3 in FPN, and N5, N4, and N3 in PAN. The specific process structure of the aggregation–distribution mechanism in yolov8 is shown in the following Figure 7.

#### 3.2.3. Loss Update

The loss value is composed of localization loss used to measure the difference between the model’s predicted bounding box and the actual bounding box, category loss used to measure the accuracy of the model’s predicted target category, and customized loss in yolov8 used to compute the difference between predicted and real feature points added together. The formula is defined as follows:

(1) $$Loss\;=\alpha_{1}Boxloss+\alpha_{2}Classloss+\alpha_{3}Dflloss.$$

$\alpha_{1},\alpha_{2},$ and $\alpha_{3}$ are weight coefficients used to balance different loss parts, BoxLoss is the boundary frame loss, ClassLoss is the classification loss, and DflLoss is the feature point loss.

The anchor-free idea is adopted in Yolov8, and Dfl loss is added in order to improve the model generalization ability. Dfl loss is a loss function used to regress the distance between the prediction box and the target prediction box. By calculating the Dfl loss, the location of the prediction box can be adjusted more accurately to improve the target detection accuracy, and the Dfl loss supervises the whole Bbox regression process to improve the target detection performance.

The same binary cross entropy loss (BCE loss) function is used in classification loss; for each sample in the binary cross entropy loss function, the difference between the predicted value and the true label is calculated and then averaged over all of its samples and the total loss value obtained. When the predicted value is exactly the same as the true label, the loss is minimum and vice versa. y is the binary label, P(y) is the probability that the output is a label, and N denotes the total number. The formula is defined as follows:

(2) $$BCEloss=-\frac{1}{n}*\sum_{i=1}^{n}y_{i}*\log p(y_{i})+\left(1-y_{i}\right)*log\left(1-p\left(y_{i}\right)\right).$$

Yolov8 uses Ciou loss in the regression task [31]. Ciou is designed to measure the degree of regression of the box by the ratio of the predicted box to the true box. However, due to the high computational complexity of Ciou, it is more sensitive to these parameters and more difficult to implement. Furthermore, because Ciou takes more information into account during the calculation, the condition that the model can learn adequately will be needed to obtain more data and facilitate a longer training time, which is a disadvantage that will be more prominent in the training of intensive images. The formula is as follows:

(3) $$Ciou=Iou-\frac{\varphi^{2}\left(b,b^{gt}\right)}{C^{2}}+\alpha *\upsilon ,$$

where

(4) $$\begin{array}{cc} \upsilon & =\frac{4}{\pi }*{\left(\arctan\frac{W^{gt}}{H^{gt}}-\arctan\frac{W}{H}\right)}^{2}.\\ \alpha & =\frac{\upsilon }{1-iou+\upsilon }, \end{array}$$

Different from this is the use of Giou’s [32] loss function as the loss used to predict the bounding box in GR-yolo. On the one hand, using Giou to obtain the weights of the predicted box and the real box in the closed region by introducing the minimum outer join matrix of the predicted box and the real box, which does not need to consider the similarity between the predicted bounding box and the real box, can help the model to converge quickly. On the other hand, Giou not only focuses on the overlapping region but also focuses on the non-overlapping region, which can better respond to the degree of overlap between the two, overcoming the problem of not being able to evaluate between overlapping and non-overlapping regions, improving the model’s prediction accuracy of the target location, and reducing the occurrence of missed detection. A is the real box, and B is the verification box. The formula is defined as follows:

(5) $$Giou=iou-\frac{\left|A\cup B-A\cap B\right|}{\left|A\cup B\right|}.$$

#### 3.2.4. GR-YOLO

**Figure 8:**
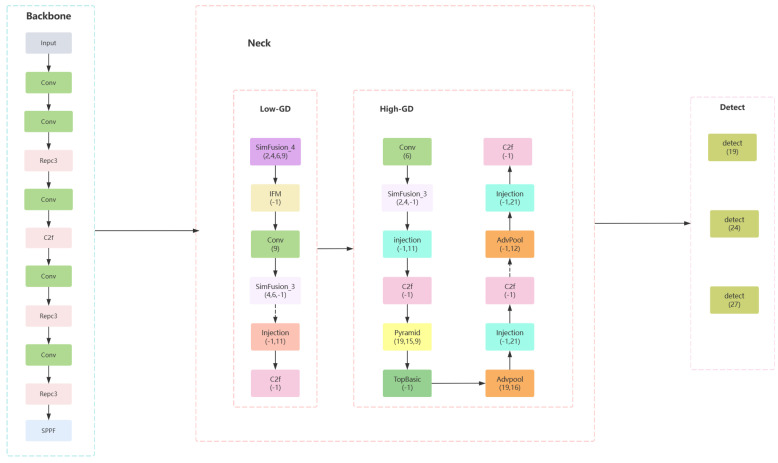
Improved GR-Yolo model structure diagram.

Based on the above improved GR-yolo, the model structure is shown below. The model structure diagram is shown in Figure 8.

In the diagram, the numbers beneath each module indicate the layer from which the input is received. Features maps of different scales are fused according to their respective levels, thereby enhancing the model’s detection capabilities. Below is a detailed introduction to some of the modules.

**Figure 9:**
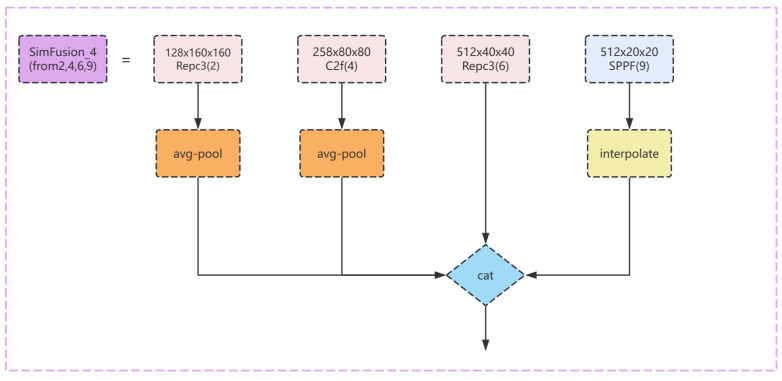
The Sim_4 module structure diagram of the improved model.

The module SimFusion_4 initially operates on four feature maps of differing scale sizes. Specifically, it performs average pooling operations on the feature maps of the first and second scales and applies bilinear interpolation to the feature map of the fourth scale. After these operations, the four feature maps are concatenated along the channel dimension, and the result is returned. The model structure diagram is shown in Figure 9.

**Figure 10:**
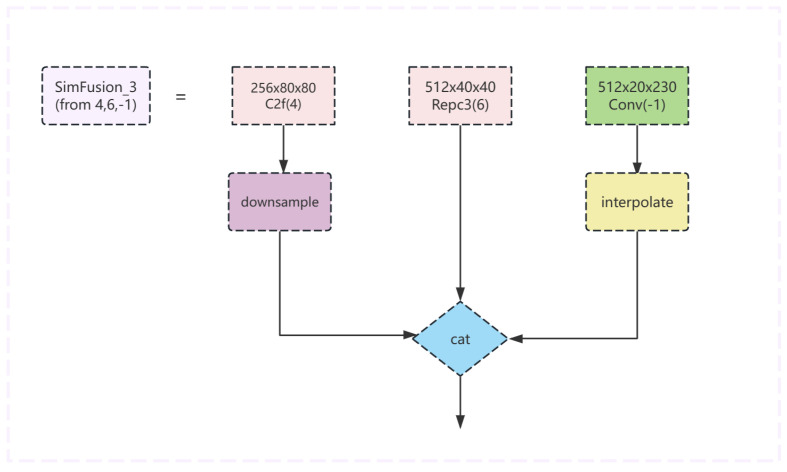
The Sim_3 module structure diagram of the improved model.

For the module SimFusion_3, the following operations are performed on three input feature maps of different scale sizes: initially, the feature map of the first part undergoes downsampling followed by a convolution operation. The feature map of the second part is subjected directly to a convolution operation. The feature map of the third part is first interpolated to a specified size before undergoing a convolution operation. Ultimately, the three processed results are concatenated along the channel dimension, fused through a fusion convolution layer, and the final output is produced. The model structure diagram is shown in Figure 10.

## 4. Experimental Part

### 4.1. Experimental Environment

**Figure 11:**
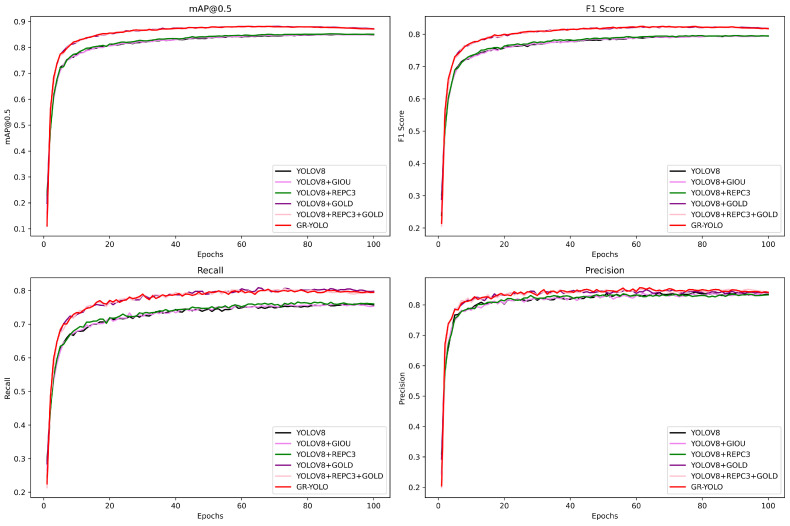
Evaluation index.

During the experiment, we trained the model several times and recorded the performance metrics of the model under different training calendar times, as shown in Figure 11 below, including precision rate, recall rate, F1 score, average mean precision, and so on. By analyzing these metrics, we found that the model’s performance gradually stabilized and reached its optimal state within 70–80 calendar hours. Specifically, we observed that the model’s performance metrics continued to improve until 70 calendar hours, while after 80 calendar hours, the model’s performance metrics began to show a steady trend. Therefore, we concluded that the model reached its optimal performance within 70–80 calendar hours; therefore, instead of adopting the official recommendation of 300 epochs by yolov8, we chose to set the epoch to 100. In order to further optimize the model’s performance, the hyperparameters were selected and adjusted based on the characteristics of the dataset and the experimental results. In the experimental validation, it was found that the effect of using the SGD optimizer was better than other optimizers. The learning rate was set to 0.01, and the kinetic energy was set to 0.937. The batch size was set to 16 to adapt to the GPU memory requirement.

Yolov10 was trained on the Pytorch 1.10.1 framework using Python version 3.9, and Python 3.8 was used to train the rest of the models presented in the paper. All experiments were executed using NVIDIA GeForce RTX 3090 GPUs (Nvidia, Santa Clara, CA, USA) on a memory size of 24,268 M. These adjustments and optimizations were made to ensure the performance of the models while improving the training efficiency and making the best use of hardware resources. The specific experimental parameter configuration is shown in Table 1.

### 4.2. Model Analysis Index

In order to evaluate the model performance more accurately, a full range of model evaluations were performed using multiple metrics such as precision, recall, F1 score, and average mean precision.

In pedestrian detection scenarios, true positive (TP) refers to pedestrians correctly identified, false positive (FP) denotes non-pedestrians incorrectly classified as pedestrians, and false negative (FN) signifies actual pedestrians that were not correctly detected.

Precision, also known as positive predictive value, refers to the proportion of correctly predicted positive observations to the total predicted positives. It is defined as follows:

(6) $$Precision\;=\frac{TP}{TP+FP}.$$

Recall, also known as sensitivity or true positive rate, measures the proportion of actual positives that the model correctly identifies. It is defined as follows:

(7) $$Recall\;=\frac{TP}{TP+FN}.$$

The F1 score is the harmonic mean of precision and recall, serving as a metric in statistical mathematics to evaluate the accuracy of binary or multi-class models. Ranging from 0 to 1, a value closer to 1 indicates better balance between precision and recall and vice versa. The definition formula for the F1 score is as follows:

(8) $$F1\;=2*\frac{Precision*Recall}{Precision+Recall}.$$

Mean average precision, commonly referred to as mAP, is the average of the average precision across all categories within all images. It represents a combination of precision and recall. For a given category, the area under the precision–recall curve is calculated, which is known as the mean average precision. mAP50 denotes the mean precision at an intersection over union (IOU) threshold of 0.5. It is defined as follows:

(9) $$Map=\frac{\sum_{i=1}^{C}AP_{i}}{C}.$$

### 4.3. Ablation Experiment

In the ablation experiments of this study, we chose the WiderPeople dataset for training and validation. This is because this dataset covers a wide range of dense pedestrians in various scenarios and can better reflect the performance of the model in dealing with pedestrian occlusion and dense scenarios. In addition, this dataset has been widely used for evaluating dense pedestrian detection models in previous studies and has a high representative and reference value.

To test the usefulness of the improved GR-YOLO for dense pedestrian detection, Yolov8, Yolov8 + Giou, Yolov8 + Repc3, Yolov8 + Gold, Yolov8 + Repc3 + Giou, and GR-yolo were used to train the WiderPeople dataset, setting up the addition of different improved ablation experiments to explore the different effects of different module improvements on the final model.

If there is no additional description, it means that the same experimental environment is set up for each group. GR-yolo is the improved yolo model proposed in this paper. Yolov8 + Repc3 is the optimization of the backbone network using the Repc3 module on top of yolov8. Yolov8 + Giou is the prediction of the bounding box using the Giou loss function on top of yolov8 for the prediction of bounding box loss. Yolov8 + Gold means using a Gold module for improvement based on yolov8. Yolov8 + repc3 + gold means using the repc3 module along with a gold module for model improvement based on yolov8. The above improvements are compared with yolov8 as a benchmark. The results of ablation experiments are shown in Table 2.

Map50 represents the average accuracy of calculating all the pictures of the pedestrian class when the iou is set to 0.5, and MAP50-95 represents the accuracy of calculating all the pictures of the pedestrian class at different IOU thresholds, ranging from 0.5 to 0.95 in steps of 0.05.

The experimental results are shown in Table 2. Comparing the experimental results in the above table, it can be seen that the GR-yolo model proposed in this paper performs well in the dense pedestrian dataset of WiderPeople, with the map50 reaching 88.1%, which is 3.2% higher compared to yolov8, and the map50-95 reaching 60%, which is 4% higher compared to yolov8. In addition, there is also an improvement in the precision rate, which is 1.9% compared to yolov8, and a significant improvement in the recall rate, which is 4% compared to yolov8. These results show that GR-yolo has significant advantages in reducing the leakage rate and improving the detection performance.

Other improvements in the module part of yolov8 in the detection of dense pedestrians were also observed: yolov8 + Giou map50 reached 85%, yolov8 was enhanced by 0.1%, map50-95 was enhanced by 0.1%, yolov8 + repc3 map50 increased to 85.2%, yolov8 map50 improved by 0.3%, map50-95 boost improved by 0.2%, yolov8 + gold map50 reached 0.879, yolov8 map50 was boosted to 3%, map50-95 was boosted 3.8%, yolov8 + repc3 + gold map50 reached 0.880, yolov8 map50 was boosted by 3.1%, map50-95 was boosted 4%, and map50-95 was boosted by 4%. All these experimental results show that the improvement aspects proposed in this paper to enhance the performance of yolov8 in dense pedestrian detection are fruitful.

Based on the results presented in the tables and images, we can see that the GR-yolo model proposed in this paper improves both precision and recall, which leads to a significant improvement in the overall performance of the detection model of yolov8. More importantly, the various modular parts that improve the yolov8 model are essential, and their interactions enable GR-yolo to maximize its benefits in dense pedestrian detection. This also illustrates that optimizing and improving model performance does not rely on a single module but requires synergy and interplay between the individual modules. With this synergy, the GR-yolo model is able to show superior performance in all situations, especially when dealing with the problem of dense pedestrian detection.

**Figure 12:**
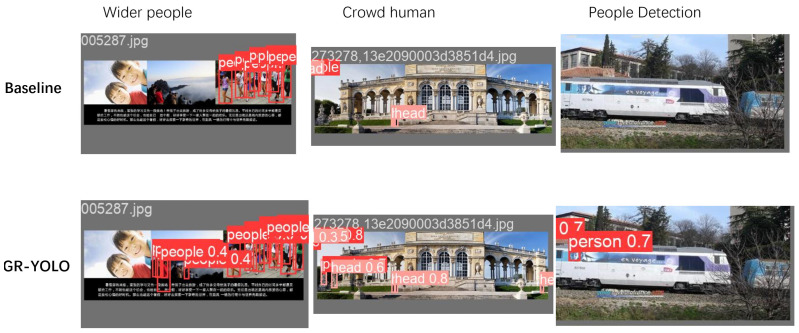
Run result.

Figure 12 shows the detection results of the original yolov8 model and our improved model, which show the comparison results in the WiderPeople dataset, CrowdHuman dataset, and People Detection dataset, respectively. From the comparison results, it can be seen that the original yolov8 model has missed detection in all three datasets, while our proposed model can better avoid missed detection. The three images are randomly selected from all three datasets, which also better demonstrates the generalization ability and robustness of our model. The results show that our adopted Repc3, Gold, and Giou modules can effectively enhance the model’s ability to detect dense pedestrians.

### 4.4. Comparative Experiment

Experimental validation was carried out using yolov5, yolov8, yolov9, yolov10, and improved GR-yolo on different datasets: WiderPeople, CrowdHuman, and People Detection, respectively. The following values are the results of map50 in the validation set. The results of comparative experiments are shown in Table 3.

From the data in Table 3, it can be seen that the GR-Yolo proposed in this paper improves on various datasets, especially on the CrowdHuman dataset, which is denser, and the improvement is very significant. GR-Yolo improved by 10.1% compared to Yolov5, 7.2% compared to Yolov8, and 4.6% compared to Yolov10.

On the generally dense WiderPeople dataset, GR-yolo improved by 3.7% compared to yolov5, 3.2% compared to yolov8, 0.1 compared to yolov9, and 4.2% compared to yolov10.

However, for the multi-scene, unevenly dense People Detection dataset, GR-yolo showed the most significant improvement. gr-yolo improved by 9.6% compared to yolov5, 2.2% compared to yolov9, 11.5% compared to yolov10, and more importantly, 11.7% compared to yolov8. This result reinforces the importance of the improvements made to the yolov8 module in this paper, especially when dealing with multi-scene, unevenly dense situations. The performance improvement of GR-yolo is particularly significant.

Yolov5 benefits from mosaic data enhancement and focus structure, performing well on all three datasets, but there is still room for improvement in the dense pedestrian detection task. By introducing C2f and adjusting the number of channels, yolov8 further improves the detection performance on the basis of yolov5 and performs better, especially in complex scenes.

The Yolov9 model is large in scale, and the larger number of layers enables it to learn more complex feature representations, which improves accuracy to some extent. However, the large scale of the model also brings some problems, such as high computational resource requirements and long training time, etc. Although Yolov10 is a new generation model, it may be affected by factors such as pedestrians occluding each other when dealing with dense pedestrian detection tasks, resulting in the difficulty of accurately detecting occluded individuals, which makes it much less effective in dense image processing scenarios. In contrast, the GR-yolo model, while maintaining a relatively small scale, effectively improves the feature extraction capability, multi-scale fusion capability, and prediction accuracy of target location by introducing the repc3 module to optimize the backbone network, adopting the aggregation–distribution mechanism to reconfigure the neck structure, as well as using Giou’s loss computation and other improvement measures, thus demonstrating higher detection accuracy and lower missed detection rate.

The GR-yolo proposed in this paper significantly improves the performance of the model in the dense pedestrian detection task. By employing several different datasets, such as the WiderPerson dataset, CrowdHuman dataset, and People Detection Image dataset, a wide variety of scenarios, crowd densities, and occlusion situations can be covered. Experiments on these datasets show that the GR-yolo model maintains good detection performance in different scenarios, demonstrating strong adaptability and robustness.

Through comparative experiments, we compared the GR-yolo model with other state-of-the-art target detection models, such as Yolov5, Yolov8, Yolov9, and Yolov10. On different datasets, the GR-yolo model outperformed the other models, especially in the dense pedestrian detection task, which fully demonstrates its robustness and superiority.

In addition, by comparing the experimental validation results of Yolov8, Yolov8 + Repc3, Yolov8 + Giou, Yolov8 + Repc3 + Giou, and GR-yolo for ablation on the WiderPeople dataset, each of the improved modules contributed positively to the performance of the model, and their interactions enabled GR-yolo to achieve higher detection accuracy and higher leakage rate in dense pedestrian detection. Yolo has higher detection accuracy and a lower miss detection rate in dense pedestrian detection. This suggests that the various parts of the model work in concert with each other to steadily improve the performance of the model.

In summary, these results show that the improved GR-yolo has higher detection accuracy and robustness in dense pedestrian detection tasks, providing strong support for future research and applications.

## 5. Results

Our work proposes a target detection model framework named GR-yolo dedicated to dense pedestrian detection. We demonstrate the superiority of GR-yolo in detection performance by performing experimental validation on the following three datasets: the large dense dataset, CrowdHuman, the large outdoor dense crowd dataset, WiderPeople, and the People Detection Images in the official roboflow released by paralytics dataset. The experimental results show that GR-yolo significantly outperforms the other comparison models in detecting these datasets, verifying its effectiveness in the dense pedestrian detection task. Although this ablation experiment mainly focuses on the WiderPeople dataset, we realize the importance of conducting ablation experiments on other datasets. In our future work, we will further expand the scope of the ablation experiments and analyze the CrowdHuman dataset and the People Detection dataset in detail in order to more comprehensively evaluate the performance of the model in different scenarios and the roles of various modules, and will try to improve the design based on these datasets and the base model, with the goal of simplifying the model structure, improving the inference speed of the model, etc. The following are some of the planned directions:

Model pruning techniques: We plan to employ state-of-the-art pruning techniques to reduce the number of parameters and computational load of the model, thereby accelerating inference speed while striving to maintain or enhance the model’s detection performance.

Innovative convolutional operations: We aim to incorporate cutting-edge convolutional operations, such as dynamic and deformable convolutions, to further improve the model’s feature extraction capabilities and detection accuracy.

Multi-task learning: By integrating other related tasks, such as pose estimation and action recognition, we seek to increase the model’s versatility and practicality.

With these improvements and optimizations, we anticipate that GR-YOLO will achieve even more outstanding performance in the field of dense pedestrian detection and provide stronger support for practical applications.

## Figures and Tables

**Table 1:** Experimental setting parameter.

Experimental Configuration	Parameters
Optimizer	SGD
Epoch	100
Learning rate	0.01
Batch-size	16
Input-size	640

**Table 2:** Ablation experimental verification.

	Precision	Recall	Map50	Map50-95
Yolov8	0.830	0.756	0.849	0.560
Yolov8 + Giou	0.832	0.757	0.850	0.561
Yolov8 + Repc3	0.833	0.765	0.852	0.562
Yolov8 + Gold	0.848	0.795	0.879	0.598
Yolov8 + Repc3 + Gold	0.849	0.796	0.880	0.600
GR-Yolo	0.855	0.796	0.881	0.600

**Table 3:** Comparative experimental verification.

	Layers	Wider People	Crowd Human	People Detection
Yolov5	214	0.844	0.741	0.707
Yolov8	225	0.849	0.770	0.686
Yolov9	930	0.880	0.872	0.781
Yolov10	385	0.839	0.796	0.668
GR-Yolo	584	0.881	0.842	0.803

## Data Availability

You can access and obtain the data through the following links: WiderHuman dataset: http://www.cbsr.ia.ac.cn/users/sfzhang/WiderPerson/ (accessed on 20 March 2024); CrowdHuman dataset: https://www.crowdhuman.org/ (accessed on 24 March 2024); People Detection Image dataset: https://universe.roboflow.com/leo-ueno/people-detection-o4rdr (accessed on 3 June 2024).
